# Annealing Gold Foil

**Published:** 1903-03-15

**Authors:** J. R. Callahan


### DENTAL REGISTER.

Vol. LVII. March 15, 1903. No. 3.

### ANNEALING GOLD FOIL.

BY J. R. CALLAHAN., D.D.S.

Read before the Ohio Dental Society, December 4, 1902.

Annealing the various forms of gold used for filling teeth, has become an interesting study to me.

In looking over the literature bearing on this subject, I have been surprised at the brief and incomplete treatment given to this subject by all the authorities consulted, except by Dr. C. N. Johnson, who, in following the teaching of Black and others, has at the same time added much that is important. To this chapter I hope to add a mite.

"We know that two pieces of perfectly pure and perfectly clean gold will weld. Two pieces of gold foil in this condition can not be rubbed together without sticking." (Dr. C. N. Johnson: *Principles and Practice of Filling Teeth.*)

If pure gold foil be exposed to the atmosphere for any length of time, or is brought in contact with certain gases, it gathers upon its surface an inperceptible film, which, while not affecting the purity of the substance itself, interferes with its cohesion. To drive off this gas and thereby restore the cohesive properties of the gold, we resort to annealing. Ordinarily this is attempted by passing the gold through an alcohol or gas flame. It is well known that we seldom have a pure alcohol or gas flame. Frequently other or additional gas films are added to the gold surface, and the latter stage is worse than the former so far as cohesion is concerned. But even if we should have a pure flame it is almost impossible to pass the gold through it in a manner that will anneal all portions of the strip or pellet, evenly. Consequently each layer of gold has different cohesive properties. A serious difficulty will be found at the extreme ends of the strip where the gold is so often melted.

When it comes to annealing pellets or crystal gold, the flame is still more objectionable. A great improvement over the flame may be obtained by arranging a platinum tray over a flame and heating the gold thereon. The only perfect heat for annealing purposes is obtained by "electrically heating a mat of coiled platinum wire. This gives a clean, uniform and accurately controlled form of heat. Electric heat differs from all other forms of heat in that it is not itself a form of combustion. It does not consume and does not emit, but radiates a pure heat." (Dr. L. E. Custer: *Dental Cosmos* for December, 1895.) So in the electric annealer we have an ideal heat for annealing purposes. This annealer, as prepared for the market, gives that degree of heat that is probably best adapted to light foils. I believe it would be a great improvement if, with the assistance of a rheostat, the annealer were capable of giving a wider range in the intensity of the heat, an improvement that can be easily made.

All authorities with which I am acquainted speak of annealing gold as if the purifying of the surface was the sole purpose of heating the foil. *To* me this is not the only purpose or result.

The miscroscopic structure of pure gold shows well defined, large crystals with sharp angles, the faces of which show different shades of color from yellow to almost black. This is probably due to the presence of small secondary crystals, apparently cubes and octahedra, for it is well known that gold crystalizes in the cubic system, being found native in this form. (*Metallography*, by Arthur H. Hirons.)

If a metal naturely assumes a crystaline structure, the molecules which are the component parts of matter, probably also assume the definite shape, and arrange themselves in contact with each other so as to form larger crystals of the same shape, and the slower and more uninterrupted the action, the larger will the crystals grow, and the more symmetrically will they arrange themselves in relation to each other, If a metal is submitted to pressure, not only will the individual crystals be distorted but the orderly arrangement of the whole mass will be disturbed, and when the pressure is carried on to a certain extent, the metal becomes rigid, a state of unstable equilibrium being brought about.

Now, in all probability the natural crystaline condition, and any deviation from this may be said to be unnatural. In such a case the molecules are ever tending to pass back again to the normal and more highly crystaline state, which is perfectly effected by re-melting and often in a great measure by annealing.

Gold, as prepared for filling purposes, comes to us in two forms, speaking broadly, in crystals and in foil. The crystal gold is a mass of minute crystals thrown down in an irregular mass by chemical means.

Foil is also a mass of crystals that have been brought together in a regular and compact or normal form by melting. This crystaline formation having been disturbed and distorted by hammering or rolling into foil. The crystals being partially re-adjusted at intervals by annealing. We have been in the habit of annealing all our gold in pretty much the same manner. That I believe to be an error which should be speedily remedied.

I believe that a little experience will show that the nearer gold is to the normal crystaline condition, the less heat it needs, and the farther the gold is from the normal crystaline condition the more protracted and the higher heat it will need for perfect results.

This can not be obtained except on an electric annealer, with a high or low heat being controlled by a rheostat.

Acting upon this belief I have tested three of the electric annealers offered for sale at the dental supply houses. The first test was with thermometers. These were not satisfactory because above 600 degrees the instrument did not behave well.

Through the courtesy of Dr. John F. Hammond, of New York, I was able to use a pyrometer—an instrument of great accuracy, with the following results. The annealers shall be known as A, B and C. The readings were taken with the covers off, for the reason that the busy dentist has enough to bother him without stopping to remove a hot cover from the annealer every few minutes.

| A | | B | | C | |
|---|---|---|---|---|---|
| MIN. | REGISTERED | MIN. | REGISTERED | MIN. | REGISTERED |
| 1 | 0° Fr. | 1. | 140° Fr. | 1 | 266° Fr. |
| 2 | 68° Fr. | 2. | 177° Fr. | 2. | 464° Fr. |
| 3. | 104° Fr. | 3. | 212° Fr. | 3. | 536° Fr. |
| 4. | 122° Fr. | 4. | 212° Fr. | 4. | 599° Fr. |
| 5. | 140° Fr. | 5. | 230° Fr. | 5 | 653° Fr. |
| 6. | 176° Fr. | 6. | 239° Fr. | 6. | 680° Fr. |
| 7. | 188° Fr. | 7. | 248° Fr. | 7. | 707° Fr. |
| 8. | 212° Fr. | 8. | 248° Fr. | 8. | 718° Fr. |
| 9. | 224° Fr. | 9. | 248° Fr. | 9. | 752° Fr. |
| 10. | 236° Fr. | 10. | 260° Fr. | 10. | 764° Fr. |

I will not claim these readings to be absolutely correct from a strictly scientific standpoint but they do show the correct relative heat-giving capacity of each instrument, and are near enough to scientific correctness for the purpose of this paper.

If the test be continued for fifteen minutes or longer, the annealers would show greater heat, but below the ten minute mark A and B showed such irregular action, and owing to the limited time at my disposal, the readings are given to the ten minute limit. At this point they were all showing rather steady progress.

In daily practice we find that each brand of gold shows an individuality that is very marked. To develop to the maximum, the cohesive properties of the various brands of gold I beg to offer the following suggestions as to degrees of heat and time of exposure.

Crystal gold about 400° for one-half minute
Soft pellets about 500° for two or three minutes.
Hard pellets about——————————————
Foil ribbons (No. 4-6) 600° for three or five minutes.
20 to 40 foils 800° for five or fifteen minutes.
60 to 120 foils 1000° for ten or fifteen minutes.
No. 60 gold and platinum, 1,000° to 1,200°' ten to twenty minutes or until the gold on the surface shows a crystaline surface.

These figures are given more to make clear the idea than to establish the exact degree of heat or time of exposure.

It will be seen that I prefer an annealer that will give a greater heat than any instrument on the market gives. I have in my office an annealer made for me by Dr. Custer that will fuse gold this being controlled by a rheostat enables me to get the greatest cohesion with any gold I desire to use, with this advantage 120 foil or 60 platinum and gold filling can be packed with the same force of blow that is required for No. 4 foil, and produce a more perfect and substantial filling.

### DISCUSSION.

Dr. J. A. Stephan: The points brought out in the paper appeal to me strongly as something practical and desirable. The usual methods of annealing gold particularly the thin forms have always appeared to me to have grave disadvantages. Undoubtedly an instrument such as is suggested by the essayist will put our foil in the best possible working form; and it seems strange that no means of controlling the degree of heat in electric gold annealers has not been called for before. I trust the essayist may continue his investigation of this subject until he shall have discovered something definite about this important matter.

Dr. W. H. Todd: I have used both alcohol and gas flames for annealing my gold, but I am confident I never got such good results as from the electric annealer, which I am using at present. It saves the operator's time as well as makes the annealing process a more perfect one. I find that fillings do not pit nor scale as when the alcohol or gas flame was used for annealing. I think the addition of a rehostat will add materially to the value of the electric annealer.

Dr. L. E. Custer: The paper read by Dr. Callahan has a good and proper basis electrically and scientifically. Gold foil, because of its treatment by the gold beaters has lost its normal crystaline character which may be restored by subjecting it to heat of the proper degree. Cohesion of gold is not possible unless there exists a normal relation of its molecular particles. Electricity is in my judgment a preferable method of annealing gold, because there is the least danger of over-heating, or contamination from the combustion products of the alcohol or gas flame. It is true that different forms of gold will need different degrees of heat and also different methods of applying the heat, Electricity has no magnetic effects on gold that would disintegrate it or destroy its cohesive properties. The rheostat will surely control the degree of heat and make it possible to anneal any foil to the best advantage.

Dr. E. C. Kirk: I would accord heartily with the views expressed by the essayist. Uniformity in annealing gold foil is an important matter and it can unquestionably be best accomplished with the electric annealer, as it gives precision and homogenity which results in a more perfectly consolidated gold filling. It is highly desirable that a more perfect appliance than the gas or alcohol flame should be found for annealing the thin gold. It is claimed by Dr. Shumway, of Massachusetts, that tin is improved for working by annealing properly. It is highly probable that insufficient attention has been given to this important subject.

Dr. H. T. Smith: Why may not a suitable thermometer be used to determine the proper degree of heat. If the heats required are no higher than indicated in the paper, I see no reason why a suitable thermometer should not provide sufficient control for all practical purposes. A thermostat might be employed were it not so expensive. It is probably true that exact temperatures are not necessary for practical results. It would seem so from the most excellent results which have come from the hands of operators who use no other methods of annealing than the gas or alcohol flames

Dr. H. L. Ambler: I have used the thermometer from my vulcanizer in testing the heat of a Custer annealer, and in this way I have found it possible to get approximate results in temperature. We must use a lower heat for thin than for heavy foils. Tin foil would oxidize or melt if the temperature is carried much beyond 150 degrees. A lower temperature would make the tin more cohesive, but at the same time it would also become more stiff.

Dr. H. F. Harvey: I use the Custer annealer with a rheostat and like it very much. I have experienced some trouble in using several different forms of gold in the same filling, as it will not all submit to the same heat and be equally pliable and tractable.

Dr. Callahan, in closing, said that the tendency was for all metals to recover their crystaline form, and as the electric annealer would produce a proper heat for hastening this process, it should always be employed. He knew of but one annealer that would carry a heat of over 400 degrees.

